# Radiologic recurrence after minimally invasive ventral hernia repair in patients with class III obesity: a single-center retrospective cohort study

**DOI:** 10.3389/jaws.2026.17051

**Published:** 2026-10-12

**Authors:** Ahsan Ahmed, Faizan Khalid, Ali Sadeghi, Marlon Rodriguez Valladares, Marlen Flores, Valeria Loja Pauta, Cesar Alas-Pineda, Cosman Camilo Mandujano

**Affiliations:** 1 Albert Einstein College of Medicine, Montefiore Medical Center, Bronx, NY, United States; 2 Facultad de Ciencias Médicas, Universidad Nacional Autónoma de Honduras, Tegucigalpa, Honduras; 3 Facultad de Medicina y Cirugía, Universidad Católica de Honduras “Nuestra Señora Reina de la Paz”, San Pedro Sula, Honduras; 4 Facultad de Ciencias Médicas, Universidad de Cuenca, Cuenca, Azuay, Ecuador; 5 Quantitative Biomedical Sciences Doctoral Program, Geisel School of Medicine, Dartmouth College, Hanover, NH, United States; 6 Department of Surgery, Montefiore Medical Center, Bronx, NY, United States

**Keywords:** hernia recurrence, minimally invasive surgery, obesity, ventral hernia repair, weight loss

## Abstract

**Background:**

Obesity is a known risk factor for complications and recurrence after ventral hernia repair (VHR). Many surgeons defer elective repair in patients with class III obesity based on body mass index (BMI) thresholds. We evaluated postoperative outcomes and factors associated with recurrence after minimally invasive VHR in this high-risk population.

**Methods:**

This single-center retrospective cohort study included consecutive adult patients with BMI ≥40 kg/m^2^ who underwent predominantly minimally invasive VHR between January 1, 2012, and December 31, 2024, and had postoperative radiologic follow-up by computed tomography (CT). The primary outcome was radiologic hernia recurrence. Secondary outcomes included 30-day complications and postoperative weight trajectory. Between-group comparisons used Fisher’s exact or χ^2^ tests for categorical variables and Welch t-test or Mann-Whitney U test for continuous variables, as appropriate; two-sided p < 0.05 was considered significant.

**Results:**

Fifty-two patients (mean BMI 45.0 ± 5.4 kg/m^2^), 80.8% female, met the inclusion criteria. Radiologic recurrence occurred in 9 patients (17.3%; CI 9.4%–29.7%) at a median imaging follow-up of 793 days (IQR 412–1381) in the overall cohort. Recurrence was observed exclusively in incisional (2/16, 12.5%) or recurrent incisional hernias (7/21, 33.3%) (p = 0.028), with no recurrences in primary hernias (0/15). 30-day complications occurred in 11.5% of patients. Patients with recurrence experienced significantly greater postoperative weight loss than those without (15.6% vs. 4.6% of total body weight; p = 0.015). Operative technique and mesh-related variables were not significantly associated with recurrence (all p > 0.25), although the study was underpowered to detect such associations.

**Conclusion:**

Minimally invasive VHR in patients with class III obesity was associated with low short-term morbidity, but the mid-term radiologic recurrence rate of 17.3% is on the higher end of published estimates and should not be characterized as low. Recurrence occurred exclusively in incisional and recurrent incisional hernias, reaching 24.3% when primary hernias were excluded; associations with technical factors and BMI were not statistically significant in this small sample. Greater postoperative weight loss was associated with recurrence, though reverse causation and ascertainment bias cannot be excluded. Given the small sample size, these findings are hypothesis-generating and warrant confirmation in larger, prospective studies.

## Introduction

Obesity in the United States continues to increase and is projected to affect 41% of the population by 2030 [1]. This trend has important implications for surgical practice, particularly in abdominal wall reconstruction. Elevated body mass index (BMI) is a well-established risk factor for ventral and incisional hernia formation, driven by increased intra-abdominal pressure, abdominal wall strain, and impaired wound healing [2, 3]. Obesity is also associated with higher rates of postoperative complications, prolonged length of stay, and hernia recurrence [4].

Management of patients with obesity undergoing ventral hernia repair (VHR) remains challenging. Many surgeons defer elective repair above specific BMI thresholds, commonly 35 kg/m^2^, despite the absence of universally accepted guidance for patients with class II-III obesity [5, 6]. This approach aims to reduce perioperative morbidity and recurrence risk; however, it may delay care in symptomatic patients. Preoperative weight loss is often difficult to achieve, and deferral of surgery has been associated with failure to undergo elective repair and increased likelihood of emergent intervention, which carries worse outcomes [7, 8].

Minimally invasive surgery (MIS) techniques have altered the risk profile of VHR and may mitigate obesity-associated morbidity. Compared with open repair, MIS approaches are associated with reduced wound complications, postoperative pain, and length of stay [9, 10]. Intraperitoneal onlay mesh (IPOM; without defect closure) and intraperitoneal onlay mesh plus (IPOM+; with defect closure) require less tissue dissection than retromuscular approaches and have demonstrated acceptable recurrence rates in selected populations [11]. However, the optimal BMI range for these approaches in patients with severe obesity remains understudied.

In this study, we evaluated postoperative outcomes following minimally invasive VHR in patients with BMI ≥40 kg/m^2^, with a focus on hernia recurrence and perioperative complications. We hypothesized that MIS VHR could be performed in this high-risk population with acceptable perioperative outcomes and explored factors potentially associated with recurrence.

## Materials and methods

### Study design and setting

We conducted a retrospective cohort study at Montefiore Medical Center, a tertiary referral academic hospital in Bronx, New York. The study was approved by the Montefiore Institutional Review Board (IRB protocol No. 2024-16476) with a waiver of informed consent due to the retrospective use of routinely collected clinical data.

### Data sources

Patients were identified from a prospectively maintained institutional hernia surgery database, with prespecified variables supplemented by structured electronic medical record abstraction when not captured in the database.

### Participants

We screened consecutive adult patients (age ≥18 years) who underwent ventral hernia repair between January 1, 2012, and December 31, 2024, with operative intent to use a minimally invasive approach (laparoscopic or robotic-assisted). Eligible patients had a primary, incisional, or recurrent ventral hernia and body mass index (BMI) ≥40 kg/m^2^ at the time of surgery, corresponding to class III obesity as defined by the World Health Organization; the terms class III obesity and BMI ≥40 kg/m^2^ are used interchangeably throughout this report. Pregnant patients were excluded.

Because the primary endpoint required radiologic confirmation, the analytic cohort was restricted to patients with at least one postoperative computed tomography (CT) study available for assessment. The clinical indication for postoperative CT (e.g., symptoms, postoperative evaluation of complications, or surveillance) and the interval from surgery to CT were recorded to contextualize the intensity of follow-up.

### Operative approach and intraoperative decision-making

All cases were planned as minimally invasive repairs. The specific technique—laparoscopic or robotic intraperitoneal onlay mesh repair with or without primary defect closure (IPOM/IPOM+), robotic enhanced-view totally extraperitoneal repair (eTEP), and other minimally invasive abdominal wall approaches—was selected by the attending surgeon based on patient anatomy, hernia characteristics, and intraoperative findings. Procedures were performed by attending surgeons within the Division of Minimally Invasive and Bariatric Surgery; operative technique, prosthetic selection, and adjunct decision-making reflect the heterogeneity of multi-surgeon academic practice. Key operative elements (defect closure strategy, mesh selection, fixation method, and adjunct procedures, including diastasis repair) were determined intraoperatively, in accordance with routine practice standards at our center. Procedures involving conversion to open or planned hybrid operations were retained in the analytic cohort and reported explicitly; these cases were classified according to the initially planned minimally invasive operative strategy at the start of the operation, and conversion/hybrid status was treated as an operative outcome descriptor rather than a randomized assignment construct.

### Variables

Baseline variables included age, sex, BMI, American Society of Anesthesiologists (ASA) class, diabetes, chronic obstructive pulmonary disease, smoking status, HbA1c, and prior bariatric surgery. Hernia characteristics were obtained from preoperative imaging when available and included hernia type, defect dimensions, and presence of rectus diastasis. Operative variables included surgical platform/approach, operative time, mesh use and dimensions, prosthetic composition, fixation method, suture type, and concomitant procedures.

### Outcomes

The primary outcome was radiologic hernia recurrence, defined as a postoperative CT finding consistent with recurrent herniation at the prior repair site (abdominal wall defect at the repair location). Three secondary outcomes were assessed. The first was the occurrence of 30-day postoperative complications, identified by chart review and comprising seroma, surgical site infection, and intra-abdominal abscess. The second was clinical recurrence, defined as a palpable or symptomatic bulge documented on physical examination. The third was time to radiologic recurrence: time-to-event was measured from the date of index repair to the date of the first CT demonstrating recurrence, and patients without radiologic recurrence were censored at the date of their most recent postoperative CT.

### Statistical analysis

Continuous variables are summarized as mean (SD) when approximately normally distributed and as median (IQR) otherwise; normality was assessed by visual inspection and the Shapiro-Wilk test. Categorical variables are summarized as counts and percentages. Comparisons between patients with and without radiologic recurrence used the Welch t-test, Mann-Whitney U test, and Fisher’s exact or χ^2^ tests as appropriate. Postoperative weight trajectory was additionally dichotomized as net loss versus maintenance or gain and compared between groups using the Fisher’s exact test. Radiologic recurrence proportion is reported with a 95% Wilson confidence interval. Missing data were handled using complete-case analysis with explicit denominators for each variable. Given the limited sample size and event count, analyses were prespecified as exploratory, and no multivariable adjustment was performed. Associations are interpreted descriptively. Two-sided P < 0.05 was considered statistically significant. All analyses were completed with the R Language and Environment for Statistical Computing, version 4.3.3.

## Results

### Study population and baseline characteristics

Between 2012 and 2024, 52 patients with BMI ≥40 kg/m^2^ underwent ventral hernia repair planned as minimally invasive and met the inclusion criteria. Mean age was 53.1 ± 13.5 years (range 29–80), 42 patients (80.8%) were female, and the mean preoperative BMI was 45.0 ± 5.4 kg/m^2^. Most patients were ASA class III (43/52, 82.7%). Diabetes mellitus was present in 24 patients (46.2%), COPD in 10 (19.2%), and active smoking in 5 (9.6%). HbA1c was available for 30 patients (57.7%) with a mean of 6.3% ± 1.7%. Fourteen patients (26.9%) had a history of bariatric surgery (Table 1).

**TABLE 1:** Baseline demographics and clinical characteristics stratified by hernia recurrence.

Variable	Overall (N = 52)	Recurrence (n = 9)	No recurrence (n = 43)	p value
Age, years, mean ± SD	53.1 ± 13.5	55.4 ± 14.0	52.6 ± 13.5	0.578
Male sex, n (%)	10 (19.2)	4 (44.4)	6 (14.0)	*0.057*
BMI, kg/m^2^, mean ± SD	45.0 ± 5.4	43.7 ± 3.7	45.3 ± 5.6	0.514
ASA classification, n (%)	​	​	​	0.139
II	7 (13.5)	3 (33.3)	4 (9.3)	​
III	43 (82.7)	6 (66.7)	37 (86.0)	​
IV	2 (3.8)	0 (0.0)	2 (4.7)	​
HbA1c, %, mean ± SD^a^	6.3 ± 1.7	5.8 ± 1.1	6.4 ± 1.8	0.717
Active smoker, n (%)	5 (9.6)	0 (0.0)	5 (11.6)	0.573
COPD, n (%)	10 (19.2)	1 (11.1)	9 (20.9)	0.670
Diabetes mellitus, n (%)	24 (46.2)	3 (33.3)	21 (48.8)	0.480
History of bariatric surgery, n (%)	14 (26.9)	2 (22.2)	12 (27.9)	1.000
Hernia classification, n (%)	​	​	​	0.028
Primary	15 (28.8)	0 (0.0)	15 (34.9)	​
Incisional	16 (30.8)	2 (22.2)	14 (32.6)	​
Recurrent incisional	21 (40.4)	7 (77.8)	14 (32.6)	​
Case classification, n (%)	​	​	​	0.420
Elective	41 (78.8)	7 (77.8)	34 (79.1)	​
Urgent	9 (17.3)	1 (11.1)	8 (18.6)	​
Emergent	2 (3.8)	1 (11.1)	1 (2.3)	​
Preoperative CT hernia width, cm^b^	5.1 ± 2.5	5.0 ± 3.0	5.2 ± 2.4	0.819
Preoperative CT hernia length, cm^c^	5.2 ± 3.5	3.7 ± 2.3	5.5 ± 3.6	0.282
CT diastasis width, cm^d^	6.4 ± 3.9	6.5 ± 2.3	6.3 ± 4.1	0.425
Diastasis present, n/N (%)^e^	39/42 (92.9)	6/7 (85.7)	33/35 (94.3)	0.708

Continuous variables expressed as mean ± SD; categorical variables as n (%). P values from the Mann–Whitney U test (continuous) or Fisher’s exact test (categorical).

^a^ Available for 30 patients (57.7%).

^b^ Available for 46 patients.

^c^ Available for 44 patients.

^d^ Available for 40 patients.

^e^ Denominator reflects patients with documented diastasis status (n = 42); 10 patients with missing data excluded.

BMI, body mass index; ASA, American Society of Anesthesiologists; COPD, chronic obstructive pulmonary disease.

Recurrent incisional hernia was the most common classification (21/52, 40.4%), followed by incisional (16/52, 30.8%) and primary hernia (15/52, 28.8%). Most cases were elective (41/52, 78.8%), with 9 urgent (17.3%) and 2 emergent (3.8%). Preoperative CT-based defect width was available for 46 patients (mean, 5.1 ± 2.5 cm) and defect length for 44 patients (mean, 5.2 ± 3.5 cm). Rectus diastasis was documented in 39 of 42 patients with available data (92.9%), with a mean CT diastasis width of 6.4 ± 3.9 cm.

### Operative details by recurrence status

A laparoscopic approach was used in 43 patients (82.7%) and the robotic approach in 9 (17.3%). IPOM+ was the predominant technique (27/52, 51.9%), followed by primary closure without mesh (9/52, 17.3%), IPOM (7/52, 13.5%), robotic eTEP (4/52, 7.7%), and robotic IPOM+ (1/52, 1.9%). Four procedures (7.7%) required conversion to open or were performed as planned hybrid operations (open repair with mesh, n = 2; transversus abdominis release, n = 1; bilateral component separation, n = 1); these cases were retained and classified according to the initially planned minimally invasive operative intent at case start, with conversion/hybrid status recorded as an intraoperative event. Median operative time was 115 min (IQR, 70–173).

Mesh was placed in 41 patients (78.8%) with a mean mesh area of 248.8 ± 160.6 cm^2^. The mesh-to-defect area ratio (mesh area divided by CT-derived defect area [width × length]) was evaluable in 34 patients, with a median of 12.1 (IQR, 4.8–18.1). Posterior sheath closure was performed in 9 patients (17.3%) and diastasis repair in 5 (9.6%). Closure suture type was documented in 48 cases (absorbable, n = 30; nonabsorbable, n = 18) and no closure suture was used in 4. Concomitant hernia repair was performed in 14 patients (26.9%) (Table 2).

**TABLE 2:** Operative details stratified by hernia recurrence.

Variable	Overall (N = 52)	Recurrence (n = 9)	No recurrence (n = 43)	P value
Approach, n (%)	​	​	​	0.330
Laparoscopic	43 (82.7)	9 (100.0)	34 (79.1)	​
Robotic	9 (17.3)	0 (0.0)	9 (20.9)	​
Technique, n (%)	​	​	​	0.771
IPOM+	27 (51.9)	6 (66.7)	21 (48.8)	​
IPOM	7 (13.5)	1 (11.1)	6 (14.0)	​
Primary closure	9 (17.3)	2 (22.2)	7 (16.3)	​
Robotic eTEP	4 (7.7)	0 (0.0)	4 (9.3)	​
Robotic IPOM+	1 (1.9)	0 (0.0)	1 (2.3)	​
Other^a^	4 (7.7)	0 (0.0)	4 (9.3)	​
Operative time, min, median (IQR)	115 (70–173)	80 (64–132)	117 (82–176)	0.256
Mesh placed, n (%)	41 (78.8)	6 (66.7)	35 (81.4)	0.378
Mesh area, cm^2^, mean ± SD^b^	248.8 ± 160.6	236.5 ± 192.3	250.9 ± 157.8	0.718
Mesh-to-defect ratio, median (IQR)^c^	12.1 (4.8–18.1)	22.9 (14.1–32.4)	11.1 (4.8–17.2)	0.295
Posterior sheath closure, n (%)	9 (17.3)	1 (11.1)	8 (18.6)	1.000
Diastasis repair, n (%)	5 (9.6)	0 (0.0)	5 (11.6)	0.573
Closure suture type, n (%)	​	​	​	0.264
Absorbable	30 (57.7)	3 (33.3)	27 (62.8)	​
Non-absorbable	18 (34.6)	5 (55.6)	13 (30.2)	​
None	4 (7.7)	1 (11.1)	3 (7.0)	​
Mesh fixation, n (%)	​	​	​	0.806
Permanent tacks	18 (34.6)	4 (44.4)	14 (32.6)	​
Absorbable tacks	10 (19.2)	2 (22.2)	8 (18.6)	​
Combined	2 (3.8)	0 (0.0)	2 (4.7)	​
None	22 (42.3)	3 (33.3)	19 (44.2)	​
Concomitant hernia repair, n (%)	14 (26.9)	3 (33.3)	11 (25.6)	0.688

Continuous variables expressed as mean ± SD, or median (IQR); categorical variables as n (%). P values from the Mann–Whitney U test (continuous) or Fisher’s exact test (categorical).

^a^ Other techniques include TAR (n = 1), open repair with mesh (n = 2), and bilateral component separation (n = 1).

^b^ Available for 41 patients with mesh placement.

^c^ Available for 34 patients with both mesh area and CT defect area data.

IPOM, intraperitoneal onlay mesh; ETEP, enhanced-view totally extraperitoneal; TAR, transversus abdominis release; IQR, interquartile range.

### Outcomes and hernia recurrence

All patients had postoperative CT follow-up, with a median interval from surgery to CT of 793 days (IQR 412–1381; range 133–3102). The 30-day complication rate was 11.5% (6/52), including seroma (4/52, 7.7%), surgical site infection (1/52, 1.9%), and abdominal wall abscess (1/52, 1.9%). Radiologic hernia recurrence occurred in 9 patients (17.3%, 95% CI, 9.4%–29.7%). Among patients with radiologic recurrence, the median interval from index repair to the CT documenting recurrence was 1208 days (IQR, 351–1694). Any radiologic recurrence (hernia or diastasis) was identified in 19 of 51 evaluable patients (37.3%). Clinical recurrence was documented in 10 of 20 patients with available physical examination data (50.0%) (Table 3).

**TABLE 3:** Postoperative outcomes and weight trajectory stratified by hernia recurrence.

Variable	Overall (N = 52)	Recurrence (n = 9)	No recurrence (n = 43)	p value
Follow-up to CT, days, median (IQR)	793 (412–1381)	1208 (351–1694)	749 (424–1359)	0.483
30-day complications, n (%)	6 (11.5)	0 (0.0)	6 (14.0)	0.574
Seroma	4 (7.7)	0 (0.0)	4 (9.3)	​
Surgical site infection	1 (1.9)	0 (0.0)	1 (2.3)	​
Abdominal wall abscess	1 (1.9)	0 (0.0)	1 (2.3)	​
Radiologic hernia recurrence, n (%)	9 (17.3)	—	—	​
Any radiologic recurrence, n/N (%)^a^	19/51 (37.3)	—	—	​
Clinical recurrence, n/N (%)^b^	10/20 (50.0)	—	—	​
Operative weight, kg, mean ± SD	119.1 ± 20.6	117.1 ± 18.0	119.5 ± 21.3	0.726
Most recent weight, kg, mean ± SD	110.6 ± 22.6	98.3 ± 16.6	113.1 ± 23.1	0.100
Weight change, kg, mean ± SD	−8.5 ± 19.3	−18.8 ± 13.4	−6.3 ± 19.8	0.017
Most recent BMI, kg/m^2^, mean ± SD	41.4 ± 8.3	36.0 ± 5.1	42.6 ± 8.4	0.010
BMI change, kg/m^2^, mean ± SD	−3.6 ± 7.6	−7.7 ± 4.3	−2.7 ± 7.9	0.015
Postoperative weight loss, %, mean ± SD	−6.3 ± 15.2	−15.6 ± 10.1	−4.6 ± 15.7	0.015
Net weight lost, n (%)^c^	33 (63.5)	9 (100.0)	24 (55.8)	0.018
Bariatric surgery after hernia, n (%)	11 (21.2)	2 (22.2)	9 (20.9)	1.000

Continuous variables expressed as mean ± SD or median (IQR); categorical variables as n (%) or n/N (%). P values from the Mann–Whitney U test (continuous) or Fisher’s exact test (categorical). Bold P values indicate statistical significance (α = 0.05).

^a^ Includes hernia and/or diastasis recurrence on CT; 1 patient without evaluable follow-up CT excluded.

^b^ Clinical recurrence assessed among 20 patients with documented physical examination follow-up.

^c^ Net weight loss defined as any reduction from operative weight to most recent follow-up measurement; compared between groups by Fisher exact test.

BMI, body mass index; IQR, interquartile range.

### Factors associated with recurrence

Hernia classification differed between groups (p = 0.028). No primary hernia recurred (0 of 15, 0%), whereas recurrence occurred in 2 of 16 incisional hernias (12.5%) and 7 of 21 recurrent incisional hernias (33.3%). When primary hernias were excluded, radiologic recurrence occurred in 9 of 37 patients with incisional or recurrent incisional hernia (24.3%). Male sex was more prevalent in the recurrence group (44.4% vs. 14.0%, p = 0.057). All 9 recurrences occurred in patients who underwent laparoscopic repair; none occurred among the 9 patients who underwent robotic-assisted repair (p = 0.330). No patient who underwent diastasis repair developed a hernia recurrence (0 of 5, 0% vs. 9 of 47, 19.1%; p = 0.573), though the small number of repairs limits inference. Neither technique (IPOM+ 22.2%, IPOM 14.3%, primary closure 22.2%; p = 0.771), posterior sheath closure (p = 1.000), suture type (absorbable vs. non-absorbable, p = 0.264), mesh fixation method (p = 0.806), nor mesh-to-defect area ratio (median 22.9 vs. 11.1, p = 0.295) demonstrated a significant association with recurrence. The 30-day complication rate was 0% in the recurrence group and 14.0% in the non-recurrence group (p = 0.574).

### Postoperative weight trajectory

Patients who developed hernia recurrence lost significantly more weight than those who did not. Mean weight change was −18.8 ± 13.4 kg in the recurrence group versus −6.3 ± 19.8 kg in the non-recurrence group (p = 0.017), corresponding to a mean percentage body weight loss of 15.6% ± 10.1% versus 4.6% ± 15.7% (p = 0.015). The most recent BMI was lower in the recurrence group (36.0 ± 5.1 vs. 42.6 ± 8.4 kg/m^2^, p = 0.010), reflecting a greater BMI reduction (−7.7 ± 4.3 vs. −2.7 ± 7.9 kg/m^2^, p = 0.015). All 9 patients with recurrence lost weight during follow-up, whereas 19 of 43 patients without recurrence (44.2%) maintained or gained weight (p = 0.018). Rates of bariatric surgery after hernia repair were comparable between groups (22.2% vs. 20.9%, p = 1.000). Operative weight did not differ between groups (p = 0.726).

## Discussion

In this cohort of patients with class III obesity undergoing minimally invasive ventral hernia repair, we observed low short-term morbidity but a radiologic recurrence rate of 17.3%, which is on the higher end of recurrence rates reported after minimally invasive VHR and should not be characterized as low. This is likely attributable to several factors specific to this cohort, including prolonged and non-standardized imaging follow-up (median 793 days, with some patients followed beyond 3 years), a high proportion of incisional and recurrent incisional hernias (71.2% of the cohort), and use of radiologic rather than clinical recurrence as the primary endpoint, which is expected to capture asymptomatic and subclinical failures that would otherwise be missed on physical examination alone. Among patients with recurrence, the median time to CT-confirmed recurrence was 1208 days (IQR, 351–1694), indicating that clinically meaningful recurrence continued to accrue well beyond the first postoperative year. In unadjusted comparisons, we did not observe statistically distinguishable associations between recurrence and individual technical variables (including defect closure, mesh fixation, or suture type); however, the limited number of events constrains precision and increases the likelihood of false-negative findings. Recurrence occurred exclusively in incisional and recurrent incisional hernias, a pattern considered further below. Greater postoperative weight loss was associated with increased recurrence; given the observational design and time-dependent follow-up, causality cannot be inferred.

Long-term outcomes after ventral hernia repair in patients with class III obesity are not well characterized. In population-based data with complete follow-up, reoperation for recurrence after elective incisional hernia repair continued to accrue throughout 5 years of observation, indicating that recurrence risk does not plateau within the first postoperative years [12]. Our median imaging follow-up of 793 days therefore reflects mid-term rather than long-term durability, and the 17.3% radiologic recurrence rate reported here is best regarded as a lower bound on what would be observed with longer, protocol-driven surveillance. Comparable long-term data restricted to patients with BMI ≥40 kg/m^2^ undergoing minimally invasive repair are, to our knowledge, not available, and this remains an important gap in the literature.

Obesity is consistently associated with worse outcomes following ventral hernia repair, particularly in open surgery, where higher complication and recurrence rates have been well documented [6, 13]. However, evidence supporting specific BMI thresholds for surgical eligibility remains limited [14]. Large registry studies demonstrate increasing risk with higher BMI, yet analyses focused on minimally invasive repair suggest attenuation of this effect [7, 15]. In an ACS-NSQIP analysis, only BMI ≥50 kg/m^2^ was independently associated with complications after minimally invasive VHR [3]. A systematic review and meta-analysis of 11 studies and 3,199 patients found no significant differences in hernia recurrence, seroma, or SSI rates among patients with BMI >40 kg/m^2^ undergoing minimally invasive VHR [16]. Similarly, Maspero et al. found that class III obesity was not associated with higher complication or recurrence rates after laparoscopic repair [2].

Recurrence was not independently associated with operative technique or any mesh-related variable; however, given the limited sample size and event count, this null finding should not be interpreted as evidence that technical factors do not influence repair durability, and larger, adequately powered studies are needed to clarify these associations. Recurrence appeared more closely associated with hernia phenotype than with the technical variables evaluated, occurring only in incisional and recurrent incisional hernias, consistent with prior evidence that defect complexity is a key determinant of failure [17, 18]. Parker et al., in a systematic review and meta-analysis of 274 studies, confirmed that recurrent hernia type is an independent prognostic factor for recurrence, above and beyond BMI alone [17].

This stepwise concentration of recurrence, from 0% in primary hernias to 12.5% in incisional hernias to 33.3% in recurrent incisional hernias, is consistent with the “vicious cycle” model of ventral hernia repair described by Holihan et al., in which postoperative complications increase the risk of recurrence and recurrence leads to further repair, with each successive operation leaving the abdominal wall more scarred, less vascularized, and structurally weaker than at the prior operation, so that each subsequent repair attempt carries a progressively higher risk of failure [19]. No 30-day complications were recorded among the patients who later developed recurrence in this cohort, so the complication-driven component of this cycle could not be observed directly; the stepwise gradient across hernia phenotypes is nonetheless consistent with its structural component. Under this framework, recurrent incisional hernia is not simply a more severe variant of incisional hernia, but a distinct risk state shaped by the cumulative physiological toll of prior surgery, independent of BMI.

No patient who underwent concomitant diastasis repair (0 of 5) developed a hernia recurrence, compared with 9 of 47 patients (19.1%) who did not undergo diastasis repair. This difference did not reach statistical significance (p = 0.573), and the small number of diastasis repairs limits any firm inference; however, the finding is mechanistically plausible and merits comment. Unaddressed diastasis may leave residual midline laxity and altered load distribution across the abdominal wall, which could plausibly place additional mechanical strain on the primary repair site over time. Considered alongside the weight-loss association described below, this observation raises the possibility that dynamic biomechanical factors of the abdominal wall, beyond the technical repair itself, contribute to recurrence in this population; this hypothesis warrants dedicated prospective evaluation.

Case acuity and hernia type also bear on the interpretation of these findings. No primary hernia recurred (0 of 15), a favorable signal; however, primary hernias in this cohort were by definition less likely to be complex or previously repaired, which likely explains this finding at least as much as any inherent protective effect of the primary phenotype itself. Among the two emergent repairs, one recurrence occurred, and among the nine urgent repairs, one recurrence occurred. These numbers are too small to support formal statistical comparison and are presented descriptively; the higher perioperative morbidity associated with emergent repair in this population is considered below.

The association between greater postoperative weight loss and recurrence is unexpected. One possible explanation is that substantial weight reduction alters abdominal wall mechanics and reduces tension across the repair, potentially destabilizing the mesh-tissue interface [20]. Prior work by Schlosser et al. demonstrated that weight change significantly impacts intra-abdominal and hernia volumes, providing a plausible mechanical basis for this observation [21]. Three alternative interpretations warrant consideration. First, reverse causation: patients with subclinical recurrence may experience altered abdominal wall integrity, caloric intake, or activity, which may contribute to weight change rather than the reverse. Second, ascertainment bias: patients who lose weight may be more likely to undergo repeated imaging, increasing the probability of detecting otherwise occult recurrence. Third, differential follow-up time and the use of the “most recent” weight/BMI (rather than a standardized timepoint) may inflate between-group differences if recurrence status correlates with duration or intensity of clinical observation. None of these mechanisms can be excluded by the present design.

Deferral of repair carries its own risks. A large NSQIP analysis showed that obese patients are disproportionately represented in emergent VHR, with complication rates nearly doubling [22]. Liu et al. identified a BMI of 35.3 kg/m^2^ as a statistically derived recurrence threshold [23], yet a recent systematic review found that BMI does not independently affect recurrence after open abdominal wall reconstruction, reinforcing the need for individualized patient selection [24]. In a cohort restricted to BMI ≥40 kg/m^2^, such thresholds should be viewed as context-setting rather than prescriptive, and decisions should remain phenotype- and risk-based.

This study has several important limitations that should temper interpretation of our findings. It is a small, single-center, retrospective series of 52 patients with only 9 recurrence events; a study of this size is not powered to support strong clinical conclusions and should be regarded as exploratory and hypothesis-generating rather than confirmatory. All comparisons are unadjusted, and the limited event count precluded meaningful multivariable modeling; we therefore cannot exclude confounding by unmeasured or imprecisely measured variables, and associations that did not reach significance in this sample (such as male sex, p = 0.057) may still reflect a true effect that this study was underpowered to detect.

Restricting inclusion to patients with postoperative imaging follow-up may have introduced selection or ascertainment bias, as clinicians may have preferentially ordered CT imaging in patients with symptoms or clinical concern for recurrence, potentially inflating the observed recurrence rate relative to the full operative cohort. In addition, all procedures were performed by attending surgeons within a single, high-volume Division of MIS and Bariatric Surgery, whose comfort and expertise in operating on patients with severe obesity and in performing complex minimally invasive abdominal wall reconstruction likely exceed that of general surgeons in most practice settings; the low short-term morbidity observed here may reflect this concentrated expertise rather than the safety of MIS VHR at this BMI threshold more broadly, and our results should not be generalized to lower-volume centers or to surgeons without comparable subspecialty training. Heterogeneity in operative technique, mesh selection, and fixation across a 12-year, multi-surgeon experience further limits the ability to draw technique-specific conclusions. The association between postoperative weight loss and recurrence should be interpreted with particular caution given the observational design, the use of “most recent” weight rather than a standardized timepoint, and the potential for reverse causation and ascertainment bias discussed above. Strengths of this study include imaging-based follow-up with explicitly reported CT intervals, use of objective radiologic recurrence rather than clinical recurrence alone, and inclusion of consecutive patients at a high-volume academic center with prospectively maintained data capture.

Several considerations follow for future study of this population. First, prospective multicenter collaboration or registry-based designs will be required to accumulate sufficient events for multivariable modeling, since single-center cohorts of this size cannot support adjusted analysis. Second, imaging follow-up should be protocol-driven and obtained at prespecified intervals rather than clinically indicated, which would remove the ascertainment bias inherent to our design and permit valid time-to-event analysis. Third, postoperative weight should be recorded at standardized timepoints so that the relationship between weight trajectory and recurrence can be assessed without the confounding introduced by variable follow-up duration. Fourth, the apparent signal associated with concomitant diastasis repair, and more broadly the contribution of abdominal wall biomechanics to repair durability, merits dedicated prospective evaluation. Finally, studies in this population should report patient-reported outcomes and long-term durability alongside recurrence, since the question facing surgeons is not only whether repair is technically feasible but whether it durably benefits the patient.

In this exploratory single-center cohort, MIS VHR in patients with class III obesity was associated with low short-term morbidity, but the mid-term radiologic recurrence rate of 17.3% should not be characterized as low, and it reached 24.3% once primary hernias were excluded. Recurrence was observed exclusively in incisional and recurrent incisional hernias; associations with the technical variables evaluated did not reach statistical significance, and this study was not powered to compare these factors against one another. Given the retrospective design, limited sample size, and small number of recurrence events, these findings should be interpreted cautiously and considered hypothesis-generating. Larger prospective multicenter studies with protocol-driven, longer-term imaging follow-up are needed to confirm these observations, to characterize long-term durability in this population, and to better define patient selection and factors associated with durable repair.

## Data Availability

The datasets presented in this article are not readily available because data access is restricted due to patient privacy regulations (HIPAA) and IRB policies of Albert Einstein College of Medicine/Montefiore Medical Center (IRB #2024-16476). Requests to access the datasets should be directed to CM camilomandujano@gmail.com.
